# An empirical study on learners’ learning emotion and learning effect in offline learning environment

**DOI:** 10.1371/journal.pone.0294407

**Published:** 2023-11-16

**Authors:** Xiangwei Mou, Yu Xin, Yongfu Song, Jinshan Xiang, Yuanbin Tang

**Affiliations:** 1 The Teachers College for Vocational and Technical Education, Guangxi Normal University, Guilin, Guangxi, China; 2 College of Electronic and Information Engineering/Integrated Circuits, Guangxi Normal University, Guilin, China; Osaka University, JAPAN

## Abstract

The application of non-cognitive factors represented by facial emotion in educational evaluation has attracted much attention in recent years. There are many existing studies on facial emotion assisted education evaluation, but most of them are based on virtual learning environments, which means that the research on facial emotion and learning effect in offline learning environments is sparse. In order to solve this problem, this study designed an emotion observation experiment based on the offline learning environment, obtained the type of learner facial emotion and learning effect of 127 college students, and further explored the relationship between the two. The results show that: 1) We obtained eight types of learner emotion through the combined description method: joy, relaxation, surprise, meekness, contempt, disgust, sadness, anxiety and their respective PAD emotional mean. 2) We obtained the correlation results of the six emotions of joy, relaxation, surprise, meekness, contempt, and anxiety with the learning effect and the predicted value of the learning effect. 3) We then constructed an explanatory model of learner emotion and learning effect based on the offline learning environment.

## 1. Introduction

Learners’ cognitive states of learning are considered to be a key determinant of learning effect and a core component of educational evaluation. However, the importance of non-cognitive factors was recognized by educational psychologists long ago. With the development of technologies, educators began to use intelligent technologies to explore the role of non-cognitive factors in educational evaluation in a deeper way, and learners’ physiological data represented by emotions began to be applied to assist teaching [1, 2]. Emotions play an important role in human learning, and that the inclusion of emotions in the metrics of instructional evaluation helps teachers’ educational decisions [3, 4], meeting the need for teacher-student emotional interaction during classroom instruction [5, 6], providing more opportunities for improving personalized instruction and achieving low-cost instructional programs [7]. The premise of emotion-assisted instruction is that emotions must be recognizable, and there have been studies in which emotions are recognized through speech recognition [8], physiological data recognition [9], body pose recognition [10], text recognition [11], facial expression recognition [12] or multimodal approach recognition for emotions [13].Facial emotions are the most direct way to detect emotional states [7], and psychologist Mehrabian noted that the expression of emotional information is equal to 7% speech + 38% voice + 55% facial expressions [14]. Experienced teachers can assess and measure the mental state of learners by observing their facial emotions [15, 16]. They can then determine whether learners are focused on what the teacher is teaching at this time, and thus further implement educational decisions.

The key to facial emotion recognition for emotion-assisted instructional decision-making lies in how to design systematic tools for facial emotion recognition [7] and exploring the relationship between facial emotion and learning effect [17]. Although most researchers use dimensional methods/models to describe emotions, such as The Pleasure-Arousal-Dominance (PAD) Emotional State Model, ultimately learner emotions are still presented in the form of categorical labels, which tends to limit the range of emotions that can actually be described. Therefore, comprehensive and effective emotion methods/models for description are of great value in facial emotion research [7, 18]. Second, facial emotion recognition integrates many emerging technologies [19, 20], such as deep learning, and hybrid technologies. The emergence of Novel coronavirus pneumonia (COVID) triggered the large-scale popularity of online education. Based on the characteristics of the online learning environment, single character and simple background, can better make use of the advantages of various emerging technologies, so the current facial emotion-assisted teaching research is mostly based on the online learning environment [1]. However, as emotion research deepens, emotional changes not only focus on learners’ attention and interest in the course, but also emphasize the social connection aspect [21, 22], while online learning environments are trapped in time and space, technology, and other elements that make it difficult to meet face-to-face communication [23, 24]. The results of relevant studies have shown that teachers and students have a heavy communication atmosphere with a single type of emotion in an online situation [25, 26], dominated by anxiety and loneliness [27, 28], and characterized by emotional unidimensionality [29, 30], where offline learning environments are more likely to achieve communication in terms of emotional interaction [31]. It is not reasonable to rely solely on findings from studies of emotions based on online learning environments to analyze offline instruction.

Therefore, the goal of this study will be: 1) To attempt to supplement the study of learner emotion in offline learning environments; 2) To design the quantitative-qualitative mixed emotional tool for description to explore types of spontaneous learner emotion in offline learning environments, and 3) To analyze the relationship between learner emotion and learning effect to provide a basis for teachers to use in implementing intelligent decision-making.

The main differences between this study and previous studies are: first, this study investigated the dynamic role of emotions in offline situation, focusing on the specific location where emotions occur and exploring the impact on learning effect. Secondly, we focused on spontaneous learner emotions. Learner emotions originate from the teacher’s real speech in the lecture rather than from the learning materials we provide that can stimulate emotions. Finally, this study will attempt to ensure the objectivity of emotion judgment by exploring and finding the consistency of multiple emotion methods/models, which will bring more scientific rigor to the process than previous studies that used only learner self-reports.

Specifically, we have formulated the following three research questions to help us in achieving the research goals:

RQ1: How can we better characterize the learner emotions in offline learning environment?
RQ2: What is the descriptive relationship between learner emotions and learning effect?
RQ3: How can we develop an explanatory model of learner emotion and learning effect?

The remainder of this study is structured as follows. Section 2 explains the literature review related to the research questions; Section 3 reports the research methods and specific experimental steps used in this study; Section 4 obtains the results of the empirical study conducted based on the 3 research questions, Section 5 discusses the outcomes and gives suggestions for future research, and finally, Part 6 draws conclusions.

## 2. Literature review

### 2.1 The emotion theories/models for description of learner facial emotions

Learning emotion refers to the emotion that occurs in a specific place and is different from general emotion, which is mainly reflected by facial emotion. Specifically, learners are less likely to have emotions such as anger and fear during the learning process, while emotions such as interest and confusion are common during the learning process [32–34].

In previous studies, emotional states were discussed under categorical and dimensional emotion theories/models. Categorical emotion models refer to the division of emotion into several independent and limited basic emotions. The basic human emotions can be divided into six categories: happiness, sadness, fear, anger, surprise and disgust. Based on this, a variety of rich emotional types can be derived [35]. Using categorical models has the advantages of being concise and easy to understand, however, the categorical labels always were limited or difficult to distinguish. This will cause great difficulties to classify facial emotion labels.

The dimensional emotion model refers to a multi-dimensional model of affects, such as The PAD Model [36]. This model uses three numerical dimensions: Pleasure, Arousal, and Dominance, to represent all emotions, where the Pleasure-Displeasure Dimension measures how pleasant an emotion may be, the Arousal-Nonarousal Dimension stands intensity of the emotion, and the Dominance-Submissiveness Dimension represents the controlling and dominant nature of the emotion [37]. The annotator can well represent emotions in three-dimensional right-angle coordinates using the Chinese version of the PAD emotion scale [38]. The various types of basic emotions are well separated in space, avoiding the use of categorical emotion models that result in inconsistent annotation and indistinguishable semantics.

The PAD model aptly combines subjective experience, external expression, and physiological arousal, which can theoretically realize the expression and quantification of all human emotions and feelings. For example, students A and B both have high P (pleasure) values in the same environment because they are both smiling, student A is very interested in what the teacher is teaching, while student B is focused on his/her own thinking about the issue. Although both students have high A (arousal) values, student A has a low D (dominance) value because he/she is controlled by the teacher, while student B has a high D (dominance) value because he/she is controlled by himself/herself, so the emotional states of the students are fundamentally different, and the learning effect is also very different. However, these two essentially different emotional states are misinterpreted as the same "joy" emotion in the categorical emotion model. Therefore, the PAD affective model undoubtedly provides a more scientific and effective tool for the study of learner affective computing.

Regarding the selection of method for description of emotion state, Wei et al. [39] defined 11 types of students’ affective behaviors by creating a list of categorical emotion labels for classroom and using the PAD labels. Wu and Lei et al. [40] selected seven important categorical facial emotion labels and evaluated the labels by the PAD model, then obtaining the features and patterns of expression interaction design for an aging intelligent companion robot. Christian et al. [41] modeled the main emotions of the virtual human in the PAD model, which was subsequently categorized into discrete emotions. Kühnlenz et al. [42] introduced an emotion adaptation method to adapt the emotional state of the robot to the emotions of the human user in the PAD model. Rincon et al. [43] constructed a dynamic emotion model for agent society based on the PAD model.

In summary, categorical and dimensional emotion theories/models have their advantages and disadvantages. Based on the intuitive visual characteristics of facial expressions, using categorical labels will be more concise and easier to understand, and combining with the PAD model helps to reduce the inconvenience caused by categorical labels due to unclear semantics, and produces good representational relationships with learners’ external expression and physiological arousal. Therefore, this study will not be limited to the use of a single emotion model for description, but will use a combined emotion model to jointly annotate learner facial emotions by exploring the consistency of multiple emotion models.

### 2.2 The relationship between learner facial emotion and learning effect

There are many databases on facial emotions, for example Compound Emotion (CE) [44], The Extended Cohn-Kanade Dataset (CK+) [45], and the Japanese Association of Female Facial Expressions (JAFFE) database [46]. However, we found that relatively few facial emotion databases were developed for learners, for example, BNU-LSVED1.0 [47] is a database developed in a real learning environment, and BNU-LSVED2.0 [39] is the first large scale spontaneous and multimodal student affect database based on the former. Generally, few databases are currently designed specifically for education or educational psychology.

Exploring the intrinsic relationship between learner emotions and learning is one of the keys to emotion-assisted instruction. Sun et al. [48] summarized learner emotional types as happy, surprised, bored, confused, fatigued, focused, and confident, and the virtual teacher responded to learners for intervention by recognizing their emotion types and screening behaviors (such as smiling, nodding, victorious, tapping shoulders, and shaking heads). Mahmoud designed an online learning platform by combining the theory of affective computing to recognize six emotions: happiness, fear, sadness, surprise, anger, and disgust [49]. Wang et al. proposed a learning fatigue recognition and intervention method based on facial emotion recognition, and suggested emotional interventions to be taken when facing learning fatigue [50].

We reviewed the relevant literature on theoretical models of the relationship between learner emotion and learning. We found that common models can be broadly classified into two types: categorical and dimensional models. The former aiming to explain the cognitive processes that elicit emotions from predefined emotions, such as The Facial Action Coding System (FACS) [51] and the Ortony, Clore and Collins Structure of Emotions (OCC) [52]. The latter viewing emotions as a combination of dimensions and learning. The well-known theory is the learning spiral model proposed by Kort, Reilly and Picard [53], which relates valence axis (positive/negative emotions) to the learning axis (learning new concepts/un-learning misconceptions) to define the learning cycle, showing the different roles of emotions in the learning process in a dynamic perspective. It however does not focus on a particular emotion [54], and has been applied only at the theoretical level. The learning spiral model provides a framework for thinking about the role of emotion in learning and is significant to the issue of emotion research in offline situations.

Based on the above related studies, we conclude that: 1. Most of the existing emotion studies originate from online learning environment, with very few studies having been specifically designed for offline situations. 2. The classification of learner emotions is mostly based on the six basic emotions, but in the face-to-face learning environments, we cannot use single or multiple discrete intensity-free nouns to characterize learners’ continuous and variable emotions. 3. Current research has shown that positive emotions are positively related to learning, while negative emotions are negatively related to learning, but it is not clear exactly where emotions occur in the learning process, and there is no operational explanatory model for the relationship between emotion and learning. Therefore, this study adopts empirical research, aiming to design an emotion research experiment for offline learning environments; proposes a combined emotion model for description; explores the types of learner emotions in offline situations; analyzes the relationship between learner emotions and learning effect; and constructs an explanatory model of emotional learning, which helps teachers make decisions and supports multi-modal educational evaluation in offline situations.

The main differences between this study and previous studies are: first, this study investigates the dynamic role of emotions in offline situation, focusing on the specific location where emotions occur and exploring the impact on learning effect. Secondly, we focus on spontaneous learner emotions. Learner emotions originate from the teacher’s real speech in the lecture rather than from the learning materials we provide that can stimulate emotions. Finally, this study attempts to ensure the objectivity of emotion judgment by exploring and finding the consistency of multiple emotion methods/models, which brings more scientific rigor to the process than previous studies that used only learner self-reports.

## 3. Research design and methodology

### 3.1 Experimental design

**Fig 1:**
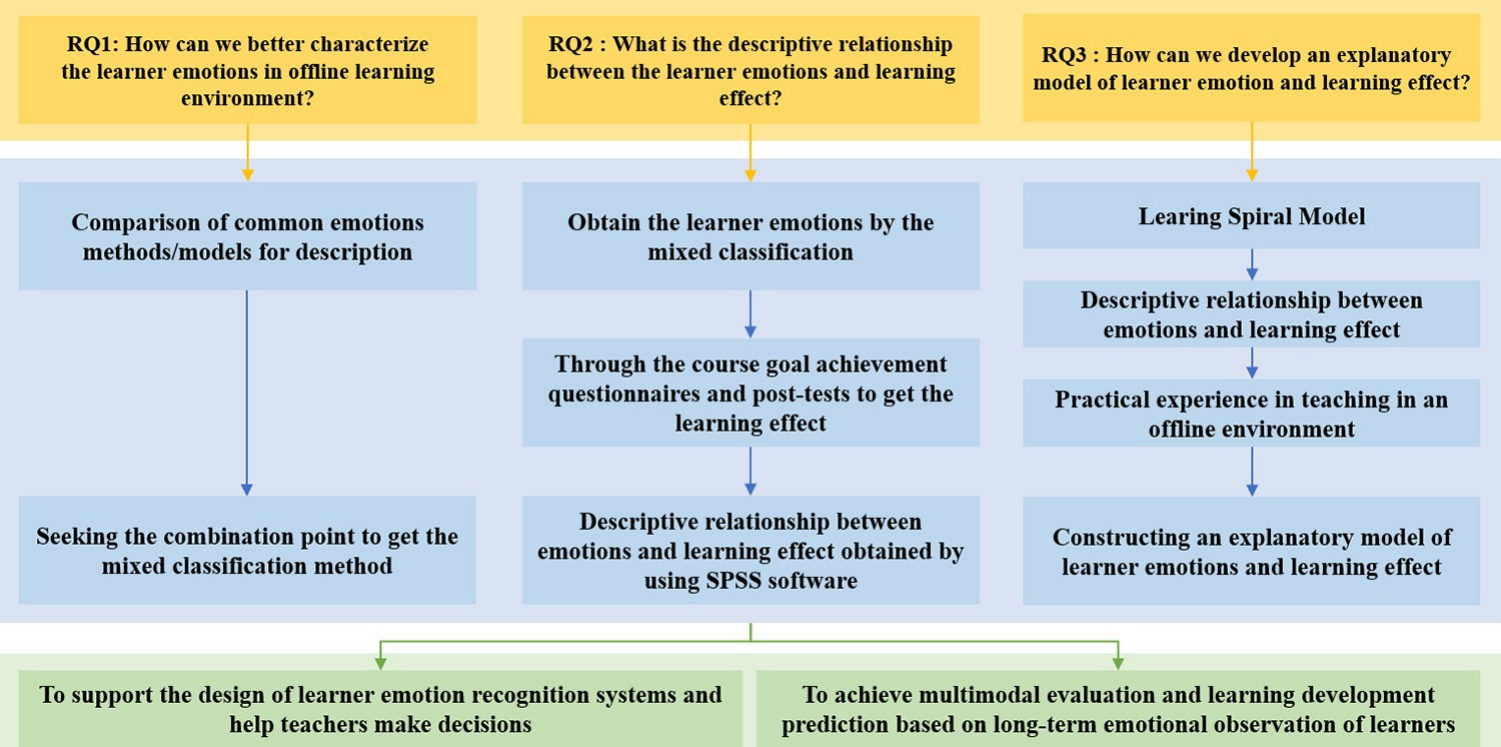
Research technology roadmap.

According to the research questions, the experimental design is determined as in Fig 1. This study adopts the empirical research to derive a combined emotion model of learner emotions to solve the problem of lacking effective tools for description of emotions, and to further explore the relationship between emotions and learning. First, we reviewed the recent relevant literature. This was done to explore the relationship between multiple emotion methods/models, and seek the combination of various emotion methods to better characterize learner emotions. Second, we designed the experiments to obtain learner emotions and learning effects, and conducted analysis to obtain a descriptive relationship between them. Third, we constructed an explanatory model of learner emotion and learning effect from the perspective of offline situations, which is based on the learning spiral model [53] and combined the descriptive relationship. It can support the design of a learner emotion recognition system to help teachers make decisions and achieve multimodal evaluation and learning development prediction based on long-term emotional observation of learners.

### 3.2 Experimental instrument

This study used a retrospective emotion judgment scheme, which has been widely used in emotion experiments [55]. The learners’ facial expressions were recorded by a camera placed in the classroom. The PAD values of emotions were obtained from the refined version of the PAD Emotion Scales, which contains three dimensions- P, A, and D-each consisting of four items. Each item consists of a word pair, which refer to feelings and highlight a special contrast between the two feelings. To facilitate learner labeling, we have modified the simplified version of the scale [38] by subdividing the set of adjectives in each item into five adjectives. For example, the first set of adjectives in the original P dimension, "Angry" and "Interested", was expanded to five adjectives, namely "Irritated", "Angry", "Neutral", "Attentive", "Interested", as shown in Table 1.

**Table 1:** Refined version of the PAD emotion scale.

Dimensionality	Item	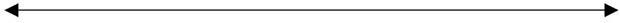													Score
		-4	-3		-2	-1		0	1		2	3		4	
P Positive and negative nature of emotions	1	Irritated		Angry			Neutral			Attentive			Interested		
	2	Friendly		Mild			Neutral			Indifferent			Contempt-ous		
	3	Painful		Troubled			Neutral			Satisfied			Delighted		
	4	Excited		Delighted			Neutral			Angry			Irritated		
A Intensity of the emotions	5	Awake		Attentive			Neutral			distracted			sleepy		
	6	Calm		Relaxed			Neutral			Interested			Excited		
	7	Interested		Attentive			Neutral			Calm			Relaxed		
	8	Indifferent		Relaxed			Neutral			Surprised			Stunned		
D The controlling and dominant nature of the emotion	9	Manipulated		Troubled			Neutral			Satisfied			Master-controlled		
	10	Dominant		Hopeful			Neutral			Accepted			Obedient		
	11	Humble		Shy			Neutral			Confident			Arrogant		
	12	Influential		Attentive			Neutral			Accepted			Influenced		

Learning effect was measured by the course goal achievement questionnaire and post-tests. The questionnaire consists of 14 questions in the form of a six-point Likert scale. We then commissioned the teachers to prepare the test questions, including fill-in-the-blanks questions, multiple-choice questions and judgment questions. To ensure the reliability and validity of the post-test, the more tenured and experienced teachers jointly developed the test.

### 3.3 Experimental environment and participants

**Fig 2:**
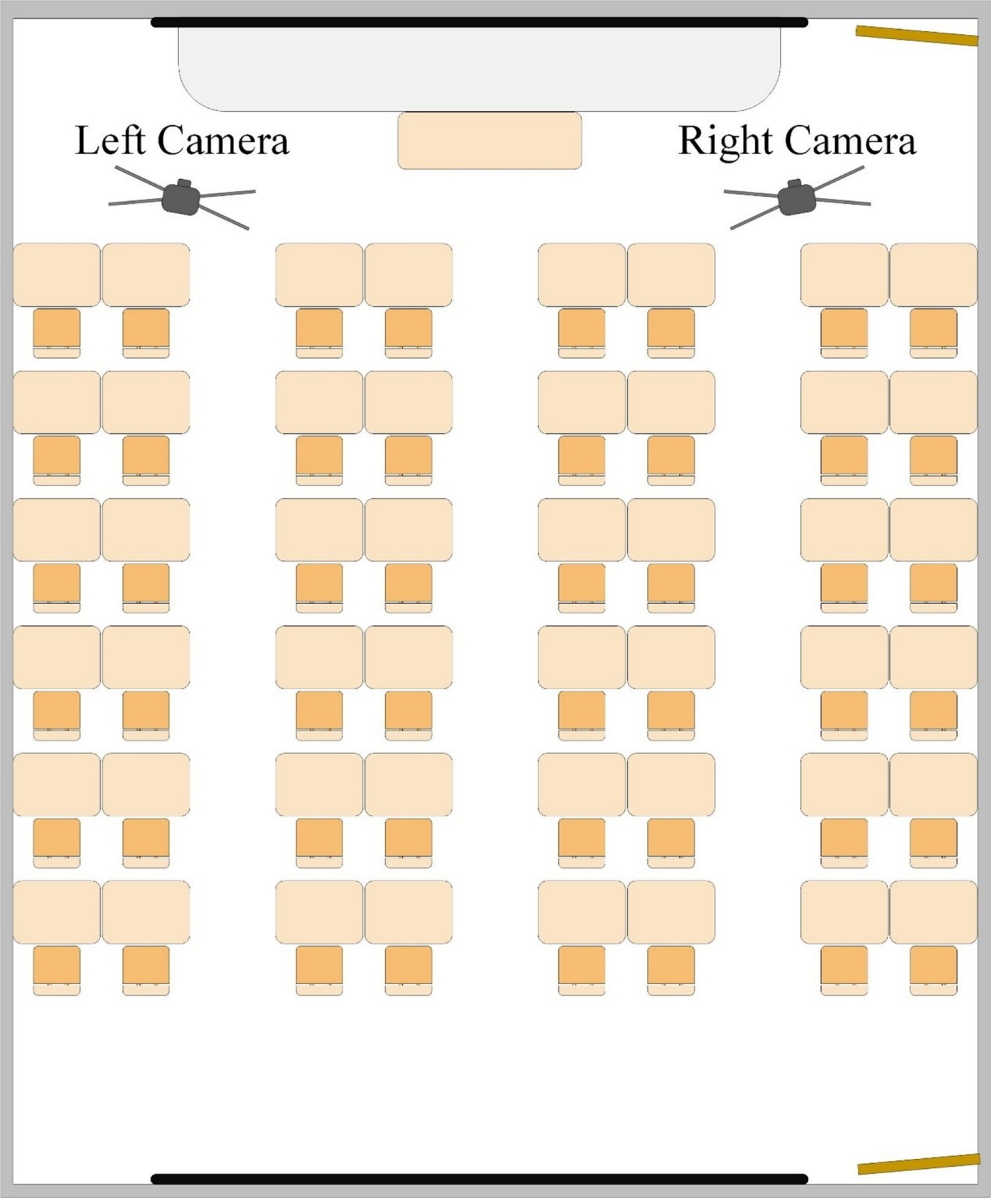
The model diagram of classroom from top view.

We selected a smart classroom at Guangxi Normal University for this study and recorded using two fixed cameras placed on either side of the classroom, as shown in Fig 2. The principle of interval seating was adopted to ensure that the facial expressions of each learner were included without obscuration. We completed the recruitment of subjects from May 1 to 15, 2022. Firstly, we published the recruitment news on the official website and WeChat public account of Vocational and Technical Teacher College of Guangxi Normal University, and then we explained the experiment purpose, clarified the experiment details and showed an informed consent form to the pre-registered subjects. The recruitment program was voluntary throughout. All experiments were approved by the Ethics Committee of Guangxi Normal University, all the details related to the experiment have been recorded, and all experimental methods have been implemented in accordance with the relevant guidelines and regulations of Guangxi Normal University, and ultimately 127 participants signed a written informed consent forms and participated in the experiment.

We also did require that the lectures be mainly theoretical lectures, and encouraged teachers to design activities that would lead students to produce more facial expressions. We eventually recorded the original 60-minute-long video, and the length of the recording did not include breaks.

In the end, we obtained 5715 facial emotion picture sequences from 127 learners. To ensure the quality of annotation, we firstly checked the distribution of each key-frames in original video. Then we checked the annotation time of all key-frames; discarded key-frames with too short time. Finally, we compared the categorical labels with the PAD values results to ensure their theoretical consistency and to avoid the situation where the labeling choice was "sadness" and the PAD values result had a positive P-value.

### 3.4 Learner facial emotion key-frame acquisition

According to the nature of objects and calculation principles, the existing research methods of facial expression recognition (FER) are mainly divided into three streams: geometric-based methods [56, 57], appearance-based methods [58, 59], and deep learning methods [56, 60]. Among them, the facial emotion recognition method based on deep learning has made rapid progress, and has shown excellent recognition effect [61, 62]. For example, Xie et al. reviewed micro-expression recognition (MER) approaches from three novel aspects: macro-to-micro adaptation, recognition based on key apex frames, and recognition based on facial action units, and then conducted research on synthetic data to solve the problem of limited and biased ME data [63]. In 2021, Monu Verma et al. designed a deep learn-based MER framework based on a perspective on promises in network model designing, experimental strategies, challenges, and research needs [64]. In the same year, the first attempt was made to discover 3-D convolutional neural network (CNN) architectures with a network-level search for MER, and proposed a novel spatiotemporal architecture search algorithm, AutoMER for micro-expression recognition (MER) [65]. In addition, in another study Monu et al. proposed an improved neural architecture search strategy to search tiny CNN architectures to find MER [66].

In short, deep learning methods have achieved great success in the computer vision area [60, 67, 68]. However, emotional feature extraction of this kind of method is relatively abstract, and the practical application cost of this technology is high, so the correlation between real classroom emotion and learning effect is not clear and direct. Geometric-based methods and appearance-based methods are primarily divided into three stages: image preprocessing, feature extraction, and feature classification [60]. In the first stage, these methods use similar preprocessing operations, the purpose of identifying and judging emotions is realized through the analysis of key frames, which effectively meets the research needs of this study, which focuses on analyzing the specific feature extraction process of learners’ faces and the relationship between emotions and learning effects, and is more conducive to practical research and application. Therefore, this study uses an emotion calibration method that conforms to the concrete image of emotion, and uses the interframe difference method to extract the emotion key frame, and the extraction platform is Windows 10 and PyCharm2021, to assist the research on the relationship between emotion and learning effect.

**Fig 3:**
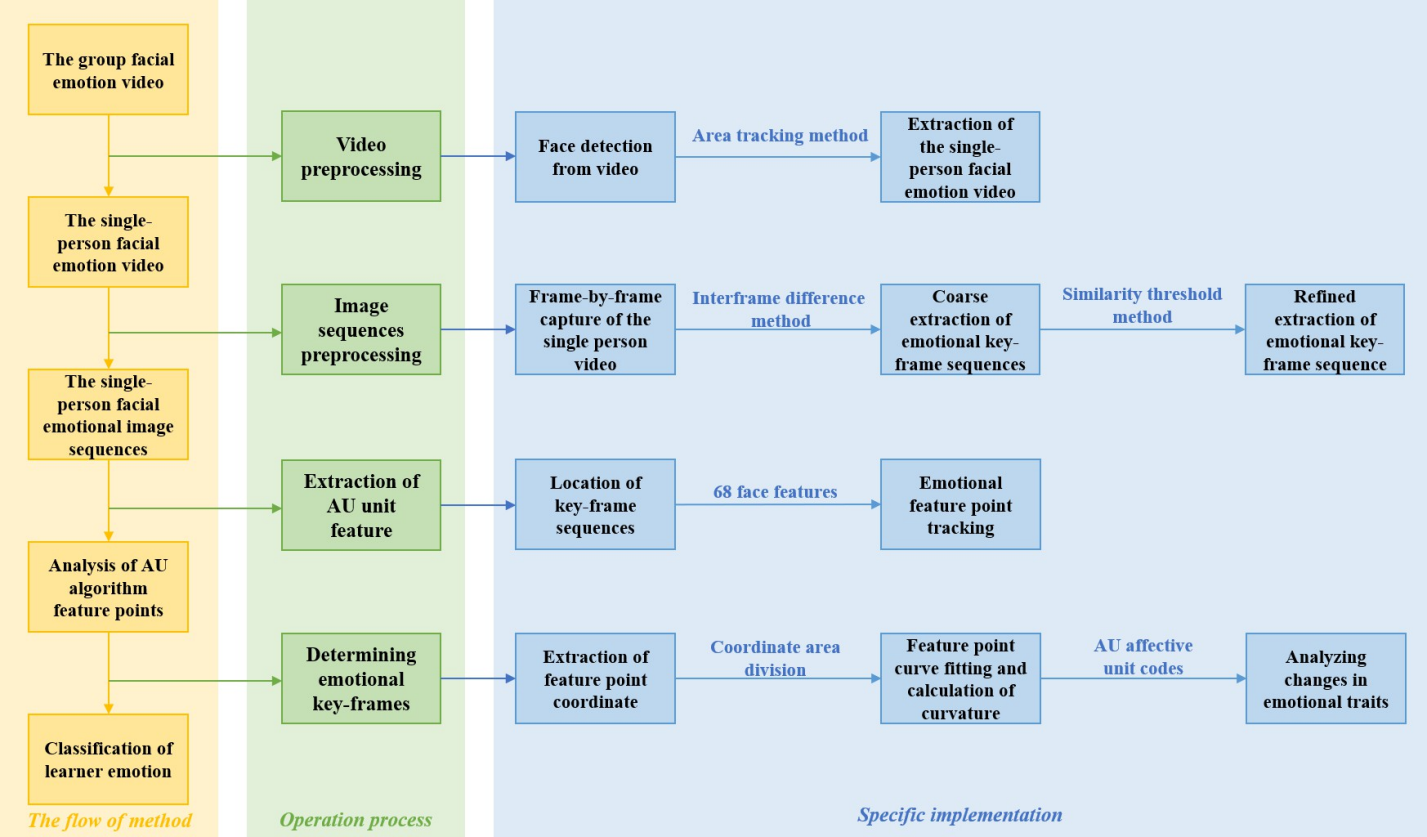
Single-person facial emotion key-frame extraction flow chart.

First, we obtained the single-person facial emotion video containing only a single face with suitable clarity by segmenting the group facial emotion video. Secondly, we captured the video stream frame by frame using the inter-frame difference method and calculated the inter-frame difference of two adjacent frames and the average inter-frame difference of all inter-frame differences. The single-person key-frame sequence was then extracted according to the principle of local maximum, which can ensure the richness of the extraction results. And the key-frames can be evenly scattered in the video, avoiding a situation where frames in similar scenes could be extracted simultaneously. Finally, AU unit feature extraction was performed on key-frames by facial feature point annotation and tracking to determine single-person emotional key-frames. The specific process is shown in Fig 3.

**Fig 4:**
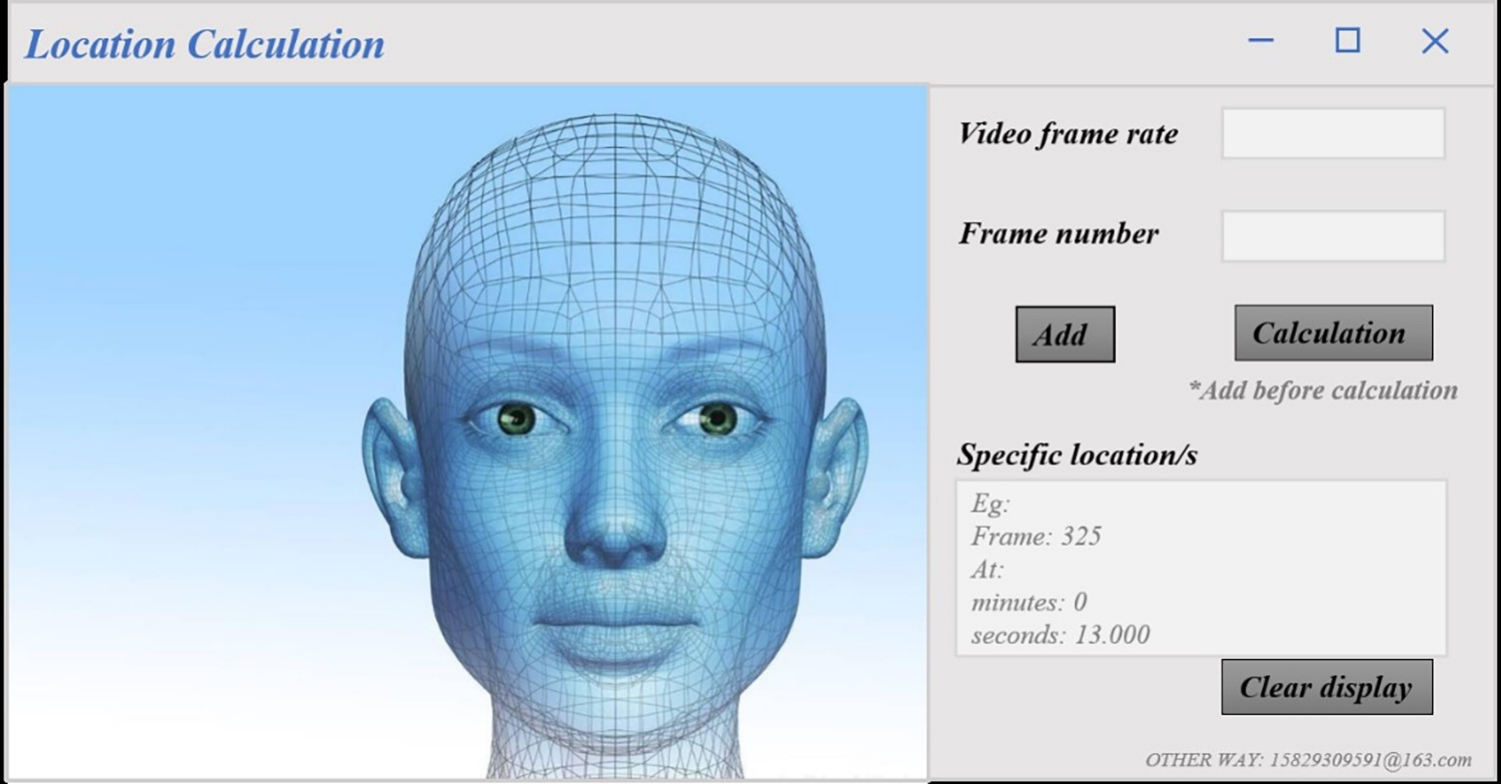
A GUI interface for batch calculation of frame sequence positions.

Because of the influence of the video background, we firstly eliminated the invalid key-frames to improve the usability of the frame sequences. Secondly, the single-person emotional key-frames were screened again by the expert group members, with the screening criterion being that when the facial expression showed strong features such as excited, or sleepy, the picture was selected as a valid emotional key-frame. Finally, we obtained no less than 30 single-person emotional key-frames of each learner, which were used for the subsequent emotions labeling. Meanwhile, to ensure the accuracy of the emotional key-frame annotation results, we deliberately created a GUI interface for batch calculation of frame sequence positions, as shown in Fig 4. Before annotating the key-frames, learners are required to watch the specific position of each key-frame in the single-person emotion video, and the before and after viewing time should be at least 5 seconds.

### 3.5 Learner facial emotion annotation

The accuracy of annotation of self-facial emotions presupposes the determination of a comprehensive and effective emotion model. We choose the categorical emotion model and the PAD emotion model together as the labeling method. This required us to explore the combination of them. Then we obtained categorical labels of learners corresponding to the range of PAD values [69]. These categorical labels included: neutral, calm, meekness, positive, joy, contempt, disgust, fear, sadness, anxiety, and angry as our list of basic emotions. We semantically interpreted and presented each label to the learners, such as neutral, representing no significant fluctuation in emotion, and calm indicating that the learner was in a relaxed state but not agile in action. We told the learners that they should select the labels we listed by recalling their own state in class. Also, we allowed learners to add new labels to enrich our basic labels. We asked learners to add new labels with as rich an explanation of the meaning of the new labels as possible. Eventually, the original list of basic emotions retained joy, meekness, contempt, disgust, sadness, and anxiety. This result that also validated the specificity of learning emotions: learners had difficulty producing strong emotions such as fear in a normal teaching environment [32]. Therefore, we removed those labels and added two new labels such as relaxation and surprise. Finally, we identified the following eight categorical emotion labels as the standard emotion labels and tentatively listed their relationships with PAD values, as shown in Table 2.

**Table 2:** Relationship between eight categorical emotion labels and PAD values.

No	Categorical emotion labels	Positive or negative attributes of PAD value
1	Joy	+P+A+D
2	Relaxation	+P−A+D
3	Surprise	+P+A−D
4	Meekness	+P−A−D
5	Contempt	−P−A+D
6	Disgust	−P+A+D
7	Sadness	−P−A−D
8	Anxiety	−P+A−D

During official labeling, we showed learners their own emotion materials, including single-person emotion videos, emotional key-frames, the refined version of the PAD scale, and a manual for using the scale. Learners were asked to read the manual and follow the steps to use the PAD scale to rate their emotional key-frames. Then we could calculate the P, A, and D values for each key-frame using the following formula, with the value of each dimension being equal to the sum of the four item scores on this dimension divided by 16 [39]. Finally, the categorical labels corresponding to the PAD values were obtained according to Table 2, so that facial emotional key-frame both had PAD values and categorical labels.


P=(Q1−Q2+Q3−Q4)/16 (1)

A=(−Q5+Q6−Q7+Q8)/16 (2)

D=(Q9−Q10+Q11−Q12)/16 (3)

## 4. Result

### 4.1 Distribution of learner emotions in offline classroom based on a mixed classification

We finally obtained 5507 valid sequences of emotion pictures through screening. A total of 4612 emotion key-frames were selected and labeled, including 663 joy, 835 relaxation, 683 surprise, 509 meekness, 560 contempt, 422 disgust, 452 sadness and 488 anxiety, as shown in Table 3.

**Table 3:** P, A and D means of the eight learner emotions.

Item	PAD values	Amou-nt	*Mean*±S.D.E.		
			P value	A value	D value
Joy	*+P+A+D*	663	0.338±0.034	0.347±0.037	0.196±0.026
Relaxation	*+P−A+D*	835	0.063±0.013	−0.340±0.034	0.014±0.003
Surprise	*+P+A−D*	683	0.285±0.034	0.386±0.042	−0.236±0.024
Meekness	*+P−A−D*	509	0.048±0.009	−0.347±0.039	−0.347±0.042
Contempt	*−P−A+D*	560	−0.210±0.024	−0.313±0.032	0.089±0.020
Disgust	*−P+A+D*	422	−0.156±0.017	0.469±0.045	0.625±0.060
Sadness	*−P−A−D*	452	−0.230±0.025	−0.262±0.030	−0.395±0.043
Anxiety	*−P+A−D*	488	−0.213±0.020	0.475±0.048	−0.3±0.030

Different types of categorical emotions correspond to distinguishable PAD values. Joy and surprise can both indicate positive and strong emotions in Chinese, but there is a distinction between active exploration and passive acceptance. In the PAD values results, the P and A values of both are positive and close, but the D values have positive and negative differences. Similarly, contempt and disgust are both negative emotions in Chinese. According to the learners’ explanation, some students will ignore the class when they are not interested in the content, while others are interested but may have difficulty understanding that the lecture. Both of the above types of learners may feel lost. However, the former is more inclined to be indifferent, while the latter would seem to express the complexity of the content. Thus, the emotions may vary greatly in the level of individual physiological activation, as well as in relation to the degree of exposure to external environmental influences. In the PAD values, the P-values are negative and close, whereas the A-values are opposite, and the D-values have a large difference in value. This also confirms the consistency between the categorical labels and PAD values used in this study.

### 4.2 The relationship between learner emotion and learning effect

Learning effect is obtained through the use of course goal achievement questionnaires and post-tests, where the values are calculated as follows.

The achievement value of the course goal achievement questionnaires = the actual score of the learner for the objective / the total score value set for the objective

The correct rate of post-test = number of correct questions / total number of questions

According to the suggestion of the teachers, we set the mathematical calculation of the learning effect as: learning effect = The achievement value of the course goal achievement questionnaires * 40% + The correct rate of post-test * 60%. Learning effect scores of 127 learners was obtained through the above calculation methods, according to different scores divided into "Fail", "Pass", "Good" and "Excellent". Finally, among the 127 learners, 8 had "Fail", 54 had "Pass", and 65 had "Good", and 0 had "Excellent". The results of the reliability test of the course goal achievement questionnaires indicated that the results were good (*Cronbach*′*s alpha coefficient* = 0.929, *KMO value* = 0.853), and the analysis of the post-test questions by teachers showed that the questions were moderately difficult and could validate the test learning effect of the learners.

In order to test the relationship between learner emotions and learning effect, we used IBM SPSS Statistics 26.0 to test a normal distribution of learning effect and learner emotions. The results showed that all eight learner emotions and learning effect followed a normal distribution (*p*>0.05). A Pearson correlation analysis was then conducted for learner emotions, with the results showing that joy, relaxation, surprise, meekness, contempt, and anxiety were significantly correlated with learning effect (see Table 4).

**Table 4:** Correlations among learning effect, joy, relaxation, surprise, meekness, contempt, disgust, sadness and anxiety(n = 127).

Variable	1 Learning effect	2 Joy	3 Relaxation	4 Surprise	5 Meekness	6 Contempt	7 Disgust	8 Sadness	9 Anxiety
1 Learning effect	1.000	0.864**	-0.629**	0.899**	-0.852**	-0.842**	-0.061	-0.085	-0.477*
2 Joy	0.864**	1.000	-0.563**	0.805**	-0.801**	-0.825**	-0.096	-0.151	-0.644**
3 Relaxation	-0.629**	-0.563**	1.000	-0.622**	0.520**	0.516**	.0289**	0.070	0.500**
4 Surprise	0.899**	0.805**	-0.622**	1.000	-0.827**	-0.806**	-0.183*	-0.031	-0.585**
5 Meekness	-0.852**	-0.801**	0.520**	-0.827**	1.000	0.835**	0.168	0.118	0.718**
6 Contempt	-0.842**	-0.825**	0.516**	-0.806**	0.835**	1.000	0.228**	0.047	0.175
7 Disgust	-0.061	-0.096	.0289**	-0.183*	0.168	0.228**	1.000	0.081	0.528**
8 Sadness	-0.085	-0.151	0.070	-0.031	0.118	0.047	0.081	1.000	0.071
9 Anxiety	-0.477*	-0.644**	0.500**	-0.585**	0.718**	0.175	0.528**	0.071	1.000

*p < .05

**p < .001.

Among them, joy (*r* = 0.864, *p* = 0.000) and surprise (*r* = 0.899, *p* = 0.000) were significantly linearly correlated with learning effect. Both of these emotions’ PAD values belong to positive and high level of physiological activation, which indicates that the more emotional engagement the learners have, the more effective learning. This is similar to Pekrun et al. [32] that the more emotional engagement, the more motivated the learner is and the better the learning effect, while if the emotional engagement is low, the more negative the learner emotions tend to be and the lower the level of physiological activation, with the learner being more likely to slack off, producing a less effective the learning effect [70].

Relaxation (*r* = −0.629, *p* = 0.000), meekness (*r* = −0.852, *p* = 0.000), and contempt (*r* = −0.842, *p* = 0.000) were significantly negative linearly correlated with learning effect. In educational psychology, research has shown that the median levels of physiological activation (A value) are most conducive to intellectual communication and arithmetic. And that negative levels physiological activation is more likely to stimulate learners’ irrelevant thoughts that are not related to the learning task, which affects learners’ rational use of learning resources [9, 71]. Generally persistent low arousal levels of emotion can lead to failure to achieve learning goals, which then may lead to a poorer learning effect [72].

Following this line of analysis, it seems that all negative emotions belong to the negative A-values and all play a negative role in learning. We found a moderate negative correlation between anxiety ((*r* = −0.477, *p* = 0.014) and learning effect, suggesting that the more anxious the emotion, the worse learning effect, with prolonged anxiety enhancing learner frustration [53, 72, 73]. However, based on the PAD values, the A-values of anxiety was positive, indicating that anxiety has a positive effect on learning effect. In related studies, Yip et al. also found that high-achieving students generally had higher levels of anxiety [74], and Gwen et al. [75] found that higher levels of anxiety predicted higher levels of learning strategy use, which confirms that not all negative emotions in the traditional cognition hinder learners’ learning. These "complex " emotions may diminish cognitive resources such as memory, leading to lower motivation and less effective learning [72, 76–78]. Yet, anxiety increases intrinsic motivation in some individuals, prompting them to devote more energy to completing complex learning tasks in order to avoid failure [79, 80]. The relationship between disgust, sadness and learning effect was not found in this study.

In order to further examine the predictive effect of emotion on learning [72], we conducted stepwise linear regression analysis with learning effects as the dependent variable and emotions such as joy, relaxation, surprise, meekness, contempt and anxiety as independent variables.

The result is shown in Table 5. From this table, it can be concluded that meekness, surprise and contempt have a significant predictive effect on learning effect, while joy, relaxation and anxiety have no significant predictive effects. The regression equation is: learning effect = 2.971–0.201* meekness +0.113* surprise -0.109* contempt. After the regression model was adjusted, the R square was 0.762. That is, meekness, surprise and contempt can explain the difference of 76.2% of learning effect. We again took learning effect as the dependent variable and meekness and contempt as the independent variables and conducted stepwise linear regression analysis. The regression equation is: learning effect = 2.424–0.205*meekness—0.161*contempt, with the adjusted R square being 0.805, indicating that meekness and contempt can predict 80.5% of the negative learning effect. We similarly took surprise as the independent variable, the regression equation is: learning effect = 0.667 + 0.292*surprise, as shown in Tables 6 and 7, and the adjusted R square was 0.655, indicating that surprise can predict 65.5% of the positive learning effect.

**Table 5:** Results of stepwise linear regression analysis 1.

	Unstandardized coefficient		Standardization coefficient	t	Sig.
	B	Standard error	Beta		
Constants	2.972	.424		7.006	.000
Meekness	-201	.043	-.460	-4.613	.000
Surprise	.113	.041	.274	2.748	.008
Contempt	-.109	.044	-.257	-2.461	.018

* Using learning effect as the dependent variable, meekness, surprise and contempt as the independent variables

**Table 6:** Results of stepwise linear regression analysis 2.

	Unstandardized coefficient		Standardization coefficient	t	Sig.
	B	Standard error	Beta		
Constants	3.735	.102		36.767	.000
Meekness	-.205	.036	-.554	-5.616	.000
contempt	-.161	.040	-.400	-4.058	.000

*Using learning effect as the dependent variable, meekness and contempt as the independent variables

**Table 7:** Results of stepwise linear regression analysis 3.

	Unstandardized coefficient		Standardization coefficient	t	Sig.
	B	Standard error	Beta		
Constants	0.667	.197		3.382	.001
Surprise	.292	.029	.814	9.902	.000

*Using learning effect as the dependent variable, surprise as the independent variable

According to the results of stepwise linear regression analysis, learner emotions that are negatively correlated with learning effects are more predictive of learning effect, which is why much of the existing emotional research has focused on managing negative emotions rather than positive emotions [7, 79, 81, 82].

### 4.3 Build an explanatory model of learner emotion and learning effect

**Fig 5:**
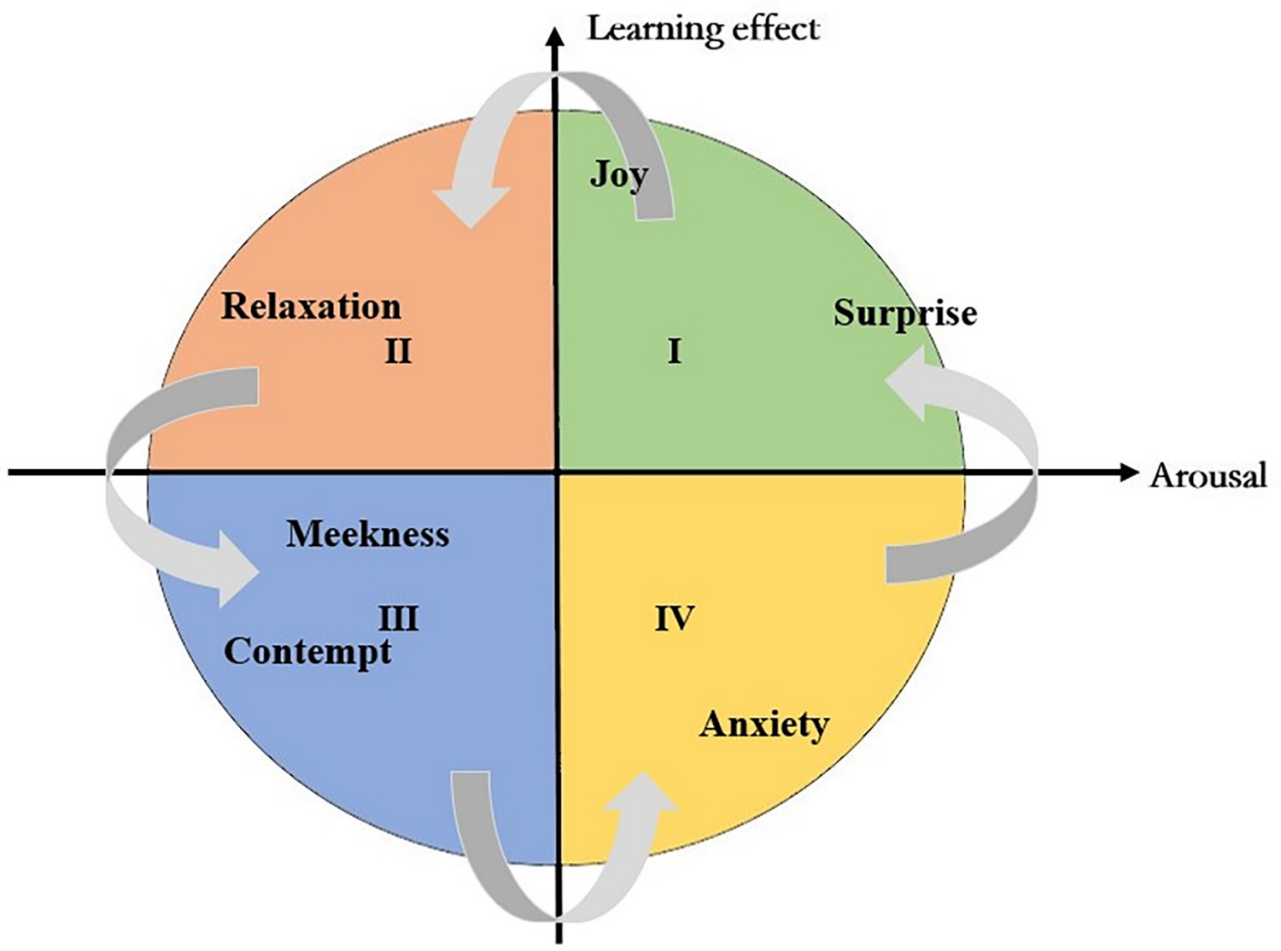
Learner emotional cycle model.

In order to better describe the relationship between learner emotion and learning effect, we tried to construct a model of the learner emotional cycle based on the results of quantitative analysis and the learning spiral model. The horizontal coordinate was defined as the arousal degree, and the positive direction indicated a higher level of learners’ emotional physiological activation. The left side indicated a lower level of emotional arousal. As shown in Fig 5. The vertical axis represented the learning effect, with the higher values more likely to produce constructive learning, better learning effect, and conversely, the poor learning effect. We arranged the eight learner emotions horizontally according to their A-values, and then vertically according to their correlation with learning effect, with the closer the emotions were to the ends of the vertical axis, the higher the correlation with the learning effect.

In actual offline learning environment, students usually start from quadrant I or II. In the beginning of the lesson, teachers always introduce the new content or review the old knowledge associated with the new. When the new situation created by the teacher greatly arouse students’ curiosity, the A value, the P value or D value tends to be mostly positive, indicating that they are interested in the teaching content (quadrant Ⅰ). Alternatively, the learners are confident in their mastery of old knowledge and feel relaxed and comfortable (quadrant Ⅱ), the A-value of learner emotion is lower at this time, but the P-value and D-value are positive, so the learning effect is at a positive status. According to the study results, we may infer that when learners in a lesson are in the upper part of the model for a long period or most of the time, the learning effect is positive.

As teaching progresses and the teacher starts to analyze the situation or teach new knowledge, the learner emotion will remain in the original state or produce new emotional changes, that is, from quadrant Ⅰ to quadrant Ⅱ or to quadrant Ⅲ. When learners are in this quadrant (Ⅲ) for a long time, we believe that learners will not learn well and teachers should pay extra attention to adjusting teaching strategies or moving to the next teaching activity to re-capture learners’ attention. Some learners will return to the upper part of the model as teachers adjust their teaching, but we also note that most learners will remain in quadrant III or move on to the next stage-quadrant IV.

The greatest change of learner emotions in quadrant IV is the fluctuation of A-values, with P-values and A-values in the negative direction or at least one of them in the negative direction. Some learners may return to the upper part of the space through self-regulation, but most of them will still be confused and lost. For example, learners with high anxiety will be motivated to study harder to eliminate this anxiety, while learners with lower levels may develop self-doubt that they cannot complete their learning tasks.

## 5. Discussion

Mixed classification is seen as a method that is more suitable for describing the diversity of emotions. However, relatively few studies are currently conducting mixed classification [7, 79–81]. According to the learner emotion statistics of this study, the mixed classification based on the positive and negative division of PAD values was found to have a good effect on emotional annotation. Each emotion key-frame has both PAD value and categorical labels, and the two labels are consistent in terms of emotion descriptiveness. However, with the refinement of PAD values, the classification of discrete emotion labels also needs to be refined. For example, when the positive and negative PAD values of emotions are the same but the mean values have obvious size differences, according to our research idea these two emotion types should correspond to the same discrete emotion label, but this is obviously unreasonable. In the follow-up research, the mixed classification still needs to explore new combinations in order to meet more emotion types needs.

The learner emotion types we obtained through the mixed classification included joy, relaxation, surprise, meekness, contempt, disgust, sadness, and anxiety, where the contempt, disgust, and sadness were consistent with the studies of Malekzadeh et al. [82] and Vogel-Walcutt et al. [83]. However, only contempt had a significant negative association with learning effect, and it had a significant predictive effect on learning effect. Another common learner emotion, anxiety, was shown to have a moderate negative association with learning effect in our study, suggesting that the effect of anxiety on learning effect is bidirectional, similar to the findings of most researchers [72, 74, 75, 79, 80]. This study summarized positive emotions such as joy and surprise, and confirmed that both joy and surprise emotions have a positive correlation with learning effect [55], while surprise has a significant predictive effect on learning effect. Therefore, future applied emotion research should shift from focusing too much on the management of negative emotions to how to promote and elicit positive learner emotions in learning situations. This study also summarized other learner emotions, relaxation and meekness, which are not common learner emotions [7]. Both had significant negative correlations with learning effect, with meekness has a significant predictive effect on learning, which was not found in previous studies. This also suggests that future emotional research should be devoted to exploring more types of learner emotions, which is a necessary prerequisite for emotion-assisted instruction.

Finally, we obtained the learner emotion types through mixed classification, and arranged these emotions into a learning-related model based on the relationship between emotions and learning effect. In the era of big data, one of the trends of future emotional research is visualization of emotions and machine analysis. In future research, we can compile emotional data into the intelligent learning platform, and analyze and obtain learner emotion values at all times through machine learning, as well as determine the emotion quadrant that learners are in. This information may prove valuable as feedback for teachers, enabling them to make real-time educational decisions, thus providing them the means to realize real-time evaluation with emotional participation. Moreover, future research can provide a means to accomplish long-term observation of learner emotions, and generate time sensitive learner emotional data reports (i.e. in terms of quarters or semesters) to realize multi-modal evaluation of learners. The emotional data report can also be used as the basis for students’ future academic analysis, personality characteristics, and career planning, offering students a personalized educational evaluation.

## 6. Conclusion

This study explored the emotion method for description and the relationship between learner emotions and learning effect in an offline learning environment through empirical study. The results showed that we obtained eight learner emotions based on mixed classification. At the same time, we further explore the relationship between these emotions and learning effects, and build a learner’s emotional cycle model based on these relationships, which contains six learner emotions in four quadrants. In this model, the learner’s emotions will change, and each quadrant’s emotions have different effects on the learning effect.

There are still some limitations in this study, and the generalizability of the findings needs to be further explored due to the small sample size and the limitations of the age, gender, and subject background of the participants. but this study provides recommendations and insights for future research directions, the findings provide a strong foundation for future research to support emotion-assisted education research.

## Supporting information

S1 Checklist STROBE statement—checklist of items that should be included in reports of observational studies. (DOCX)

S1 File (DOCX)

S1 Data (XLSX)
