# Supplementary material for: An empirical study on learners’ learning emotion and learning effect in offline learning environment
Source: PLoS One. 2023 Nov 16;18(11):e0294407. doi: 10.1371/journal.pone.0294407 (PMC10653466; doi:10.1371/journal.pone.0294407)
Supplement: S1 Checklist — (DOCX) [file pone.0294407.s001.docx]

STROBE Statement—checklist of items that should be included in reports of observational studies

|  | Item No. | Recommendation | Page  No. | Relevant text from manuscript |
| --- | --- | --- | --- | --- |
| **Title and abstract** | 1 | (*a*) Indicate the study’s design with a commonly used term in the title or the abstract | P1 | A process model of learning effect and learner emotion occurrence in offline learning environments—an empirical study for the learning spiral model |
|  |  | (*b*) Provide in the abstract an informative and balanced summary of what was done and what was found | P1 | In order to solve this problem, this study designed an emotion observation experiment based on the offline learning environment, obtained the type of learner facial emotion and learning effect of 127 college students, and further explored the relationship between the two. The results show that: 1) We obtained eight types of learner emotion through the combined description method: joy, relaxation, surprise, meekness, contempt, disgust, sadness, anxiety and their respective PAD emotional mean. 2) We obtained the correlation results of the six emotions of joy, relaxation, surprise, meekness, contempt, and anxiety with the learning effect and the predicted value of the learning effect. 3) We then constructed an explanatory model of learner emotion and learning effect based on the offline learning environment. |
| Introduction | | | |  |
| Background/rationale | 2 | Explain the scientific background and rationale for the investigation being reported | P4 | The key to facial emotion recognition for emotion-assisted instructional decision-making lies in how to design systematic tools for facial emotion recognition (Elaheh et al., 2019) and exploring the relationship between facial emotion and learning effect (Tan et al, 2021). |
| Objectives | 3 | State specific objectives, including any prespecified hypotheses | P5 | Therefore, the goal of this study will be: 1) To attempt to supplement the study of learner emotion in offline learning environments; 2) To design the quantitative-qualitative mixed emotional tool for description to explore types of spontaneous learner emotion in offline learning environments, and 3) To analyze the relationship between learner emotion and learning effect to provide a basis for teachers to use in implementing intelligent decision-making. |
| Methods | | | |  |
| Study design | 4 | Present key elements of study design early in the paper | P12 | This study adopts the empirical research to derive a combined emotion model of learner emotions to solve the problem of lacking effective tools for description of emotions, and to further explore the relationship between emotions and learning. |
| Setting | 5 | Describe the setting, locations, and relevant dates, including periods of recruitment, exposure, follow-up, and data collection | P13;P14;P15;P16 | We selected a smart classroom at Guangxi Normal University for this study and recorded using two fixed cameras placed on either side of the classroom.  We planned to recruit 127 healthy Guangxi Normal University undergraduates, with equal proportions of males and females, from different majors in the Vocational and Technical Teacher College of Guangxi Normal University.  We eventually recorded the original 60-minute-long video, and the length of the recording did not include breaks.  The learners’ facial expressions were recorded by a camera placed in the classroom. The PAD values of emotions were obtained from the refined version of the PAD Emotion Scales.  Learning effect was measured by the course goal achievement questionnaire and post-tests. |
| Participants | 6 | (*a*) *Cohort study*—Give the eligibility criteria, and the sources and methods of selection of participants. Describe methods of follow-up  *Case-control study*—Give the eligibility criteria, and the sources and methods of case ascertainment and control selection. Give the rationale for the choice of cases and controls  *Cross-sectional study*—Give the eligibility criteria, and the sources and methods of selection of participants | P15 | We planned to recruit 127 healthy Guangxi Normal University undergraduates, with equal proportions of males and females, from different majors in the Vocational and Technical Teacher College of Guangxi Normal University. To ensure the authenticity of the emotions in the spontaneous environment, we allowed individual differences among students. |
|  |  | (*b*) *Cohort study*—For matched studies, give matching criteria and number of exposed and unexposed  *Case-control study*—For matched studies, give matching criteria and the number of controls per case |  |  |
| Variables | 7 | Clearly define all outcomes, exposures, predictors, potential confounders, and effect modifiers. Give diagnostic criteria, if applicable | P21;P23 | Then we could calculate the P, A, and D values for each key-frame using the following formula, with the value of each dimension being equal to the sum of the four item scores on this dimension divided by 16.  The achievement value of the course goal achievement questionnaires = the actual score of the learner for the objective / the total score value set for the objective  The correct rate of post-test = number of correct questions / total number of questions |
| Data sources/ measurement | 8* | For each variable of interest, give sources of data and details of methods of assessment (measurement). Describe comparability of assessment methods if there is more than one group | P21;P23;P24 | According to the suggestion of the teachers, we set the mathematical calculation of the learning effect as: learning effect = The achievement value of the course goal achievement questionnaires * 40% + The correct rate of post-test * 60%.  We finally obtained 5507 valid sequences of emotion pictures through screening. A total of 4612 emotion key-frames were selected and labeled, including 663 joy, 835 relaxation, 683 surprise, 509 meekness, 560 contempt, 422 disgust, 452 sadness and 488 anxiety. |
| Bias | 9 | Describe any efforts to address potential sources of bias |  |  |
| Study size | 10 | Explain how the study size was arrived at |  |  |

Continued on next page

| Quantitative variables | 11 | Explain how quantitative variables were handled in the analyses. If applicable, describe which groupings were chosen and why | P24 | In order to test the relationship between learner emotions and learning effect, we used IBM SPSS Statistics 26.0 to test a normal distribution of learning effect and learner emotions. The results showed that all eight learner emotions and learning effect followed a normal distribution (p>0.05). A Pearson correlation analysis was then conducted for learner emotions, with the results showing that joy, relaxation, surprise, meekness, contempt, and anxiety were significantly correlated with learning effect (see Table 4). |
| --- | --- | --- | --- | --- |
| Statistical methods | 12 | (*a*) Describe all statistical methods, including those used to control for confounding | P24;P27;P28;P29 | A Pearson correlation analysis was then conducted for learner emotions, with the results showing that joy, relaxation, surprise, meekness, contempt, and anxiety were significantly correlated with learning effect (see Table 4).  In order to further examine the predictive effect of emotion (Pekrun, Elliot, & Maier, 2009) on learning, we conducted stepwise linear regression analysis with learning effects as the dependent variable and emotions such as joy, relaxation, surprise, meekness, contempt and anxiety as independent variables.  We again took learning effect as the dependent variable and meekness and contempt as the independent variables and conducted stepwise linear regression analysis.  We similarly took surprise as the independent variable… |
|  |  | (*b*) Describe any methods used to examine subgroups and interactions |  |  |
|  |  | (*c*) Explain how missing data were addressed |  |  |
|  |  | (*d*) *Cohort study*—If applicable, explain how loss to follow-up was addressed  *Case-control study*—If applicable, explain how matching of cases and controls was addressed  *Cross-sectional study*—If applicable, describe analytical methods taking account of sampling strategy |  |  |
|  |  | (*e*) Describe any sensitivity analyses |  |  |
| Results | | | | |
| Participants | 13* | (a) Report numbers of individuals at each stage of study—eg numbers potentially eligible, examined for eligibility, confirmed eligible, included in the study, completing follow-up, and analysed |  |  |
|  |  | (b) Give reasons for non-participation at each stage |  |  |
|  |  | (c) Consider use of a flow diagram |  |  |
| Descriptive data | 14* | (a) Give characteristics of study participants (eg demographic, clinical, social) and information on exposures and potential confounders |  |  |
|  |  | (b) Indicate number of participants with missing data for each variable of interest |  |  |
|  |  | (c) *Cohort study*—Summarise follow-up time (eg, average and total amount) |  |  |
| Outcome data | 15* | *Cohort study*—Report numbers of outcome events or summary measures over time |  |  |
|  |  | *Case-control study—*Report numbers in each exposure category, or summary measures of exposure |  |  |
|  |  | *Cross-sectional study—*Report numbers of outcome events or summary measures |  |  |
| Main results | 16 | (*a*) Give unadjusted estimates and, if applicable, confounder-adjusted estimates and their precision (eg, 95% confidence interval). Make clear which confounders were adjusted for and why they were included | P21—P33 | 4. Result  4.1 Distribution of learner emotions in offline classroom based on a mixed classification  We finally obtained 5507 valid sequences of emotion pictures through screening. A… |
|  |  | (*b*) Report category boundaries when continuous variables were categorized |  |  |
|  |  | (*c*) If relevant, consider translating estimates of relative risk into absolute risk for a meaningful time period |  |  |

Continued on next page

| Other analyses | 17 | Report other analyses done—eg analyses of subgroups and interactions, and sensitivity analyses |  |  |
| --- | --- | --- | --- | --- |
| Discussion | | | | |
| Key results | 18 | Summarise key results with reference to study objectives | P32;P33;P34 | According to the learner emotion statistics of this study, the mixed classification based on the positive and negative division of PAD values was found to have a good effect on emotional annotation.  However, only contempt had a significant negative association with learning effect, and it had a significant predictive effect on learning effect. Another common learner emotion, anxiety, was shown to have a moderate negative association with learning effect in our study…  we obtained the learner emotion types through mixed classification, and arranged these emotions into a learning-related model based on the relationship between emotions and learning effect. |
| Limitations | 19 | Discuss limitations of the study, taking into account sources of potential bias or imprecision. Discuss both direction and magnitude of any potential bias | P35 | There are still some limitations in this study, and the generalizability of the findings needs to be further explored due to the small sample size and the limitations of the age, gender, and subject background of the participants. but this study provides recommendations and insights for future research directions, the findings provide a strong foundation for future research to support emotion-assisted education research. |
| Interpretation | 20 | Give a cautious overall interpretation of results considering objectives, limitations, multiplicity of analyses, results from similar studies, and other relevant evidence | P34 | Both had significant negative correlations with learning effect, with meekness has a significant predictive effect on learning, which was not found in previous studies. This also suggests that future emotional research should be devoted to exploring more types of learner emotions, which is a necessary prerequisite for emotion-assisted instruction. |
| Generalisability | 21 | Discuss the generalisability (external validity) of the study results | P33 | Another common learner emotion, anxiety, was shown to have a moderate negative association with learning effect in our study, suggesting that the effect of anxiety on learning effect is bidirectional, similar to the findings of most researchers (Pekrun et al., 2002; Zeidner, 2014; Shute et al., 2015; D'Mello et al., 2014; Muis et al., 2021; Gwen, 2015; Yip, 2007). |
| Other information | |  | | |
| Funding | 22 | Give the source of funding and the role of the funders for the present study and, if applicable, for the original study on which the present article is based | P36 | This research was funded in part by The Natural Science Project of Guangxi Normal University (No.2021JC012), and in part by Youth Fund for Humanities and Social Sciences Research of the Ministry of Education (No.21XJC880004). |

*Give information separately for cases and controls in case-control studies and, if applicable, for exposed and unexposed groups in cohort and cross-sectional studies.

**Note:** An Explanation and Elaboration article discusses each checklist item and gives methodological background and published examples of transparent reporting. The STROBE checklist is best used in conjunction with this article (freely available on the Web sites of PLoS Medicine at http://www.plosmedicine.org/, Annals of Internal Medicine at http://www.annals.org/, and Epidemiology at http://www.epidem.com/). Information on the STROBE Initiative is available at www.strobe-statement.org.
