# Supplementary material for: An empirical study on learners’ learning emotion and learning effect in offline learning environment
Source: PLoS One. 2023 Nov 16;18(11):e0294407. doi: 10.1371/journal.pone.0294407 (PMC10653466; doi:10.1371/journal.pone.0294407)
Supplement: S1 File — (DOCX) [file pone.0294407.s002.docx]

Dear classmates:

In order to examine the degree of completion of the teaching objectives of the course "Control System of Industrial Robots" by the 19th grade Mechanical Design manufacture and Automation Major (Vocational Teacher Qualifications), we invite you to answer this questionnaire. This questionnaire is filled out in real-name form. The results of the questionnaire are only used for investigation and research, and will not have any impact on you. Thank you for your support and cooperation.

1. Your personal information:

Name:

Gender:

Student number:

1. I can grasp the structural contents of the industrial robot control system and its specific meaning.

□1 strongly disagree □2 relatively disagree □3 slightly disagree □4 A little agree □5 relatively agree □6 totally agree

1. I can understand the concepts and functions of industrial robot control system, operating system, drive system.

□1 strongly disagree □2 relatively disagree □3 slightly disagree □4 A little agree □5 relatively agree □6 totally agree

1. I can compare the advantages and disadvantages of the three mainstream control systems for industrial robots.

□1 strongly disagree □2 relatively disagree □3 slightly disagree □4 A little agree □5 relatively agree □6 totally agree

1. I can identify the control system block diagram of typical industrial robots including ABB, KUKA robots and some domestic industrial robots.

□1 strongly disagree □2 relatively disagree □3 slightly disagree □4 A little agree □5 relatively agree □6 totally agree

1. I can compare and understand the concepts and principles of industrial robot servo, force, vision control and integrated control system.

□1 strongly disagree □2 relatively disagree □3 slightly disagree □4 A little agree □5 relatively agree □6 totally agree

1. I can find a reasonable motion control scheme for industrial robots by consulting literature or related research.

□1 strongly disagree □2 relatively disagree □3 slightly disagree □4 A little agree □5 relatively agree □6 totally agree

1. I can try to solve the problems related to the industrial robot control system by using methods such as group cooperation through the study in the last class.

□1 strongly disagree □2 relatively disagree □3 slightly disagree □4 A little agree □5 relatively agree □6 totally agree

1. I can independently complete the post-class test questions based on the knowledge of the industrial robot control system learned in the last class.

□1 strongly disagree □2 relatively disagree □3 slightly disagree □4 A little agree □5 relatively agree □6 totally agree

1. I can use the method of summarizing to sort out and consolidate the knowledge learned in the last class, and improve the practical ability of self-discovery and problem-solving.

□1 strongly disagree □2 relatively disagree □3 slightly disagree □4 A little agree □5 relatively agree □6 totally agree

1. I can try to explore the choice of industrial robot control methods in different fields through knowledge transfer.

□1 strongly disagree □2 relatively disagree □3 slightly disagree □4 A little agree □5 relatively agree □6 totally agree

1. I have developed a sense of responsibility and mission of a powerful country in science and technology through the study of typical application cases of industrial robots.

□1 strongly disagree □2 relatively disagree □3 slightly disagree □4 A little agree □5 relatively agree □6 totally agree

1. I have established a sense of standards and norms, and strengthened my professional ethics and professional norms education through the study of the complex principles of industrial robot control systems.

□1 strongly disagree □2 relatively disagree □3 slightly disagree □4 A little agree □5 relatively agree □6 totally agree

1. I feel the practical application value of various typical industrial robots (ABB, KUKA), and have the motivation and interest to learn more.

□1 strongly disagree □2 relatively disagree □3 slightly disagree □4 A little agree □5 relatively agree □6 totally agree
